# Appendiceal Adhesion to the Gallbladder Detected During Laparoscopic Cholecystectomy: A Case Report

**DOI:** 10.7759/cureus.20625

**Published:** 2021-12-22

**Authors:** Zaki Busbaih, Jawad Busbaih, Ahmad Odeh, Abdulqader M Albeladi, Ali A Almohammed Saleh

**Affiliations:** 1 General Surgery, Prince Saud Bin Jalawy Hospital, Al-Ahsa, SAU; 2 Gastroenterology, King Fahad Hospital of Hofuf, Al-Ahsa, SAU; 3 General Surgery and Laparoscopic Surgery, Prince Saud Bin Jalawy Hospital, Al-Ahsa, SAU; 4 General Surgery, King Faisal University, Al-Ahsa, SAU

**Keywords:** laparoscopic cholecystectomy, laparoscopic appendectomy, acute appendicitis, gallstone cholecystitis, appendicular adhesion

## Abstract

Acute cholecystitis is a very common acute abdominal disease that mostly indicates abdominal surgery. Appendiceal adhesion to the gallbladder is a very rare condition detected during laparoscopic surgery. A 54-year-old female patient, with a known case of diabetes and hypertension, presented with right upper quadrant abdominal pain of four months’ duration. The pain was increasing in severity and associated with fatty meals. She was diagnosed with acute cholecystitis and was sent to the operating room for laparoscopic cholecystectomy. Appendiceal adhesion to the gallbladder was found, and laparoscopic cholecystectomy and laparoscopic appendectomy were performed. Adhesion of the appendix to the gallbladder should be considered by general surgeons.

## Introduction

Acute calculous cholecystitis (ACC) is the third most common surgical emergency worldwide [1]. ACC is considered one of the most common surgical diagnoses, and its incidence increases with age [2]. Another very common surgical emergency is acute appendicitis that plays a major role in hospital emergencies and should be considered in the differential diagnosis of patients with abdominal pain [3]. Immediate diagnosis of acute appendicitis is crucial because, when the diagnosis is delayed or if patients leave the hospital with an incorrect diagnosis, it may lead to many clinical complications such as perforation or intra-abdominal abscess [4]. The appendix is considered the most common organ that has variations in its anatomical position [5]. Therefore, knowing the anatomical variations of the appendix is necessary to avoid possible iatrogenic complications caused by appendectomy. Although both conditions (ACC and acute appendicitis) are very common, a simultaneous occurrence of both conditions is extremely rare [6]. Furthermore, there is a lack of literature that shows the adhesion of the appendix to the gallbladder. Here, we report a case of adhesions between the appendix and the gallbladder detected during laparoscopic cholecystectomy in a 54-year-old female patient.

## Case presentation

**Figure 1:**
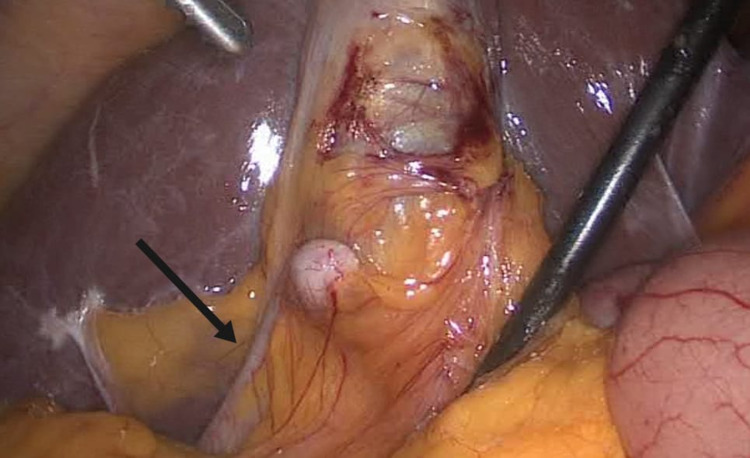
Adhesion between the appendix and the gallbladder.

A 54-year-old female patient, with a known case of diabetes and hypertension, visited a general surgery clinic complaining of abdominal pain of four months’ duration. The abdominal pain was described as a sharp pain localized to the right upper quadrant area. The pain started gradually and was increasing in severity. It was intermittent, did not radiate anywhere, and was associated with fatty food. There were no nausea or vomiting, no history of jaundice, and no diarrhea or constipation. She is neither a smoker nor an alcoholic. The patient does not have any allergies. She has a history of lipoma on the back that was surgically removed one year ago. On physical examination, the patient was conscious and oriented with a normal temperature, pulse rate of 70 beats/minute, respiratory rate of 20 breaths/minute, blood pressure of 138/71 mmHg, and oxygen saturation of 100%. General examination showed normal respiration with bilateral air entry and normal heart sounds. On focused abdominal examination, the abdomen was soft and lax with right upper quadrant tenderness. Murphy’s sign was negative. The patient was admitted as a case of ACC. An ultrasound of her abdomen revealed thickened-wall, inflamed, distended gallbladder (more than 5 mm), small multiple stones, and pericholecystic fluid. Her laboratory results were all normal, including blood count, liver function test, and kidney function test. The patient was diagnosed with ACC and has been sent to the operating room for laparoscopic cholecystectomy. Surgery was performed under general anesthesia with the patient in the supine position. Two adhesions were found during surgery. The first one is adhesion between the gallbladder and the omentum. The second one is adhesion between the appendix and the gallbladder (Figure 1).

**Figure 2:**
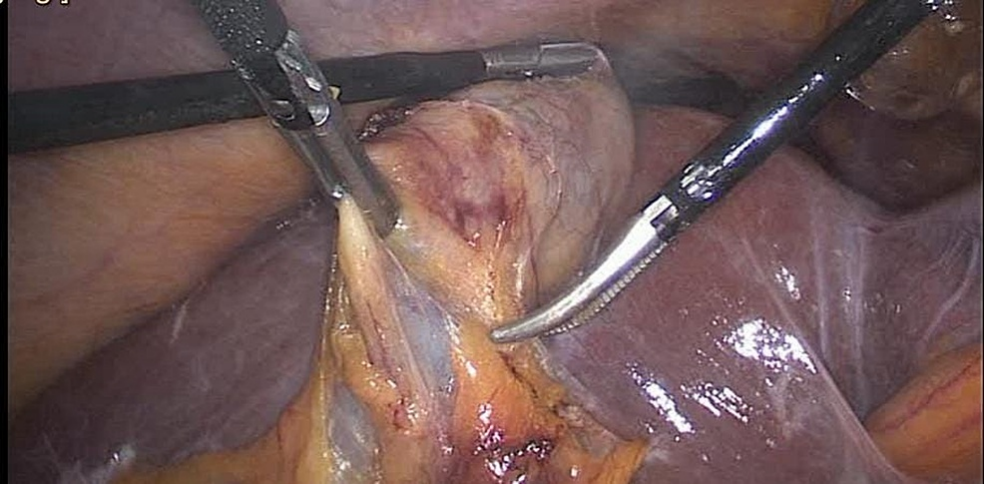
Inflamed gallbladder.

**Figure 3:**
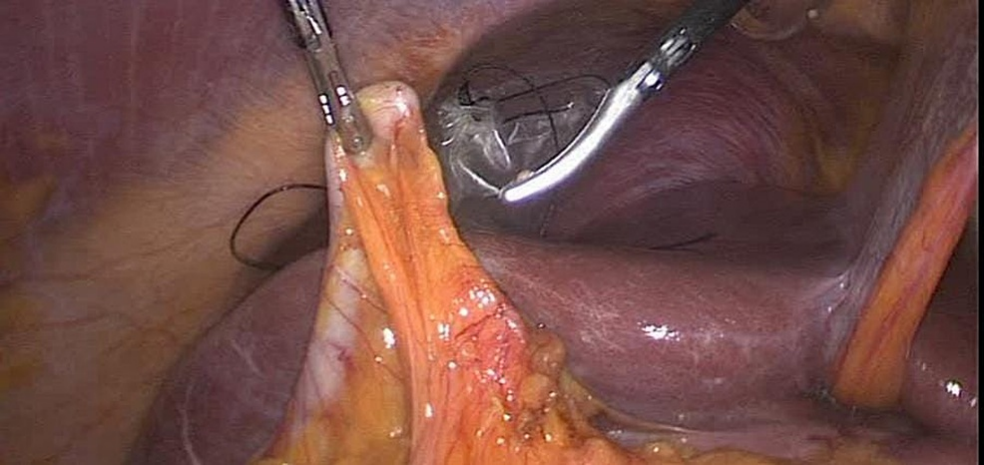
Laparoscopic appendectomy.

Firstly, the omentum adhesion has been removed from the gallbladder. Then, the appendix has been separated from the gallbladder using a ligature. After completion of cholecystectomy (Figure 2), adhesiolysis using a harmonic device was performed. The cystic duct and artery were clipped. The gallbladder was dissected from the liver bed. Laparoscopic appendectomy (Figure 3) was performed, and hemostasis was secured. The specimen was then processed for histopathology. Gross description of the gallbladder revealed an opened gallbladder (7 × 3 cm). The maximum wall thickness was 0.3 cm. The serosal surface was shiny and unremarkable. The mucosa was pale green. The appendix was 9 cm in length × 1 cm in diameter. Regarding microscopic description, the gallbladder wall was seen with focal intestinal metaplasia and chronic cholecystitis. It was negative for dysplasia. The appendix was histologically unremarkable. Postoperatively, the patient was improving and tolerated a soft diet. There were no complaints of pain. The abdomen was soft and lax with clean wounds. The patient was discharged home on postoperative day one and made an uneventful and full recovery.

## Discussion

This report illustrates the possibility of appendiceal adhesion to unusual organs and unexpected locations. Older and comorbid patients may present with a nontypical presentation where the diagnosis may be reached only during surgery [7,8]. Appendiceal adhesion to the gallbladder is an extremely rare condition that reports in the literature regarding such a case were lacking. In the emergency department, acute cholecystitis and acute appendicitis are considered very common acute conditions [9]. The history of right upper quadrant abdominal pain and Murphy’s sign are strongly suggestive of acute cholecystitis, but it is less frequent in older adults [10]. The diagnosis of ACC in our patient was confirmed by an ultrasound scan after suspecting it clinically. Generally, cholecystectomy is considered the definitive management that can be performed [11]. It is safe, plausible, and fast, so our patient was in full recovery without further treatment needed. In patients with a retrocecal appendix, complaints might vary since it imitates right upper quadrant and right flank diseases such as acute cholecystitis, diverticulitis, and renal colic, making diagnosis challenging [12]. However, patients with acute appendicitis typically complain of initial paraumbilical pain that migrates to the lower quadrant [13]. In our patient, the presentation was more suggestive of ACC rather than acute appendicitis as pain and tenderness were more prominent at the right upper quadrant and the ultrasound image showed multiple small size stones and thickened-wall gallbladder. In the literature, there were some case reports of synchronous presentation of acute appendicitis and cholecystitis [14-16]. Demuro reported a case of synchronous acute cholecystitis and acute appendicitis that was managed laparoscopically [17]. Moreover, Sahebally et al. described a case of a male patient who complained of epigastric abdominal pain where his abdominal ultrasound result showed an inflamed, thickened-walled gallbladder with no evidence of stone. The appendix was not visible. Appendiceal inflammation was revealed during laparoscopy [18]. Biliary reflux or gallbladder dyskinesia associated with acute appendicitis that was relieved after an appendectomy was described by Carter et al. [19]. Previously, it has been noted that hyperbilirubinemia could occur in acute appendicitis [20]. The synchronous occurrence of acute appendicitis and cholecystitis is explained as a result of bacterial translocation into the portal venous system, leading to altered bilirubin excretion [10]. However, appendiceal adhesion to the gallbladder with normal or abnormal bilirubin levels has not been reported. We believe that the reason behind this appendiceal adhesion to the gallbladder in our patient is congenital as there was no evidence of appendicitis.

## Conclusions

Adhesion of the appendix could occur in many ways. However, adhesion of the appendix to the gallbladder is rarely seen. Thus, even in the absence of abnormal laboratory results, surgeons should be aware of these types of variations to avoid unfamiliar complications. As we reported in this case, appendiceal adhesion was found during laparoscopic cholecystectomy.
